# Cancer chemoprevention through Frizzled receptors and EMT

**DOI:** 10.1007/s12672-021-00429-2

**Published:** 2021-09-09

**Authors:** K. Sompel, A. Elango, A. J. Smith, M. A. Tennis

**Affiliations:** Division of Pulmonary Sciences and Critical Care Medicine, School of Medicine, University of Colorado Anschutz Medical Campus, 12700 E 19th AVE, RC2 Box C272, Aurora, CO 80045 USA

**Keywords:** Frizzled, Chemoprevention, Cancer, Epithelial to mesenchymal transition

## Abstract

Frizzled (FZD) transmembrane receptors are well known for their role in β-catenin signaling and development and now understanding of their role in the context of cancer is growing. FZDs are often associated with the process of epithelial to mesenchymal transition (EMT) through β-catenin, but some also influence EMT through non-canonical pathways. With ten different FZDs, there is a wide range of activity from oncogenic to tumor suppressive depending on the tissue context. Alterations in FZD signaling can occur during development of premalignant lesions, supporting their potential as targets of chemoprevention agents. Agonizing or antagonizing FZD activity may affect EMT, which is a key process in lesion progression often targeted by chemoprevention agents. Recent studies identified a specific FZD as important for activity of an EMT inhibiting chemopreventive agent and other studies have highlighted the previously unrecognized potential for targeting small molecules to FZD receptors. This work demonstrates the value of investigating FZDs in chemoprevention and here we provide a review of FZDs in cancer EMT and their potential as chemoprevention targets.

## Background

Frizzled (FZD) receptors, belonging to the superfamily of G-protein coupled receptors (GPCR) Class F, have a major role in development and tissue homeostasis in humans. There are ten FZD receptors, all containing the classical seven pass transmembrane residues and a highly conserved N-terminus cysteine-rich domain [1]. FZD receptors bind to glycoprotein Wingless/Int (WNT) ligands, in conjunction with various co-receptors, such as low-density lipoprotein receptor related proteins (LRP) 5/6, receptor tyrosine kinase like orphan receptor 2 (ROR2), receptor-like tyrosine kinase (RYK), and vanglike-1 (VANGL-1), forming a heterodimeric complex. Formation of this complex activates many downstream signaling targets in both canonical and non-canonical pathways [2]. In the canonical pathway, FZDs control β-catenin signaling by promoting nuclear translocation of β-catenin when activated by a WNT ligand. Once a WNT ligand binds to a FZD receptor it forms a complex with LRP5/6 and the disheveled (DVL) protein, enabling the accumulation of β-catenin in the cytoplasm [3]. Cytoplasmic β-catenin translocates to the nucleus, binds to the T-cell factor/lymphoid enhancer factor (TCF/LEF) family of transcription factors, and upregulates the transcription of multiple WNT-associated genes related to cell stemness, proliferation, apoptosis, polarity, motility and differentiation [4]. FZD receptors also regulate cellular processes through non-canonical signaling. The planar cell polarity (PCP) pathway regulates tissue or cytoskeleton organization and maintenance through VANGL and DVL [5]. WNT/Ca^2+^ signaling regulates intercellular Ca^2+^ signaling through the Diacylglycerol/Inositol Trisphosphate (DAG/IP3) pathway [6]. In the past, non-canonical and canonical WNT signaling were considered mutually exclusive, but recent literature suggests there is more overlap between them [7].

Initially FZD receptors in cancer were described only as part of oncogenic β-catenin signaling, but some FZDs, along with a variety of WNT ligands and co-receptors, have also been identified as tumor suppressive [8, 9]. FZD receptors have low mutation rates in cancer, with no more than 10% of patients in any cancer type having any FZD mutation and most mutations appearing in less than 2% of patients [10]. Despite the lack of common mutations, dysregulation of WNT/FZD signaling pathways leads to tumorigenesis through increased cell proliferation, epithelial to mesenchymal transition (EMT), and invasion [11],. Alterations in FZD mRNA and protein expression play a role in regulating EMT. Many events take place in the transition of an epithelial cell to a mesenchymal cell, including dissociation of cell–cell junctions, transition to front-rear cell polarity, and reorganization of the cytoskeleton [12]. EMT is characterized by down regulation of epithelial genes e-cadherin and zonula occludens–1 and upregulation of mesenchymal genes snail, slug, n-cadherin, vimentin and fibronectin [13]. These changes can occur at the earliest stages of lesion development and serve to eventually enhance tumor cell motility and invasion. Several of the pathways that are involved in EMT are also regulated by the β-catenin canonical and non-canonical pathways. For example, normal epithelial cells need to maintain strong cell–cell contacts; this is led by e-cadherin, with the support of β-catenin, α-catenin and actin cytoskeleton. When the canonical pathway is activated, β-catenin does not bind to e-cadherin, which leads to disassociation of cell-to-cell contacts [14]. The non-canonical PCP pathway alters cytoskeletal organization and the Wnt/Ca^2+^ pathway is associated with cancer cell motility and invasion, all of which relate to EMT processes [14, 15]. Increased understanding of the role of FZDs in EMT could lead to new prevention approaches for high-risk populations and recent studies suggest renewed interest in FZD receptors as druggable targets [16, 17].

The complex mutational profile and microenvironment of a mid- or late-stage tumor can make treatment complicated and allow for multiple pathways of drug resistance. Chemoprevention in high-risk populations intercepts progression of premalignant lesions to invasive tumors and reduces cancer deaths [18]. Early lesions, though often not visible by imaging, can be identified by changes in markers such as Krt5, Krt14, vimentin and n-cadherin, and by transcription factors such as TRP63, GRHL1-3, ZEB1/2, TWIST1/2, and SNAI1 [19]. Many of these markers are also mechanistically associated with EMT processes. Recent examples of approaches explored for cancer chemoprevention that target EMT include metformin, silibinin, β-carotene, and celecoxib [20–23]. The efficacy of chemopreventive drugs may depend on expression of targeted receptors, as with FZD9 and the lung cancer chemoprevention drug iloprost [8, 24, 25]. The role of FZD receptors in EMT and the influence of EMT on progression of premalignant lesions suggests that FZD receptors may be appealing targets for chemoprevention. This review discusses the role of individual FZD receptors in cancer EMT and the potential for FZD receptors to be cancer chemoprevention targets.

## Frizzleds in cancer EMT

### Frizzled 1

Formerly known as, *Drosophila FZD* or FZD wingless receptor, FZD1 is located on chromosome 7q21 [26]. FZD1 is expressed in adult hippocampus, where knocking down FZD1 impaired neuronal differentiation and but does not affect dendritic development [27]. Frizzled 1 receptors are moderately expressed in most adult tissues, with high expression levels observed in bone, lung, small intestine, liver and female reproductive tissues [28, 29]. Within the FZD receptor family, FZD1 shares the most homology with FZD2 and FZD7 [30]. The known WNT ligands for FZD1 are WNT1, WNT8, WNT3, WNT3a, and WNT2 [31]. FZD1 is most notably known for its regulation of Drosophila wing development and the PCP pathway. FZD1 acts as an oncogenic regulator; when expression is down regulated an overall decrease in tumorigenesis is observed in both neuroblastoma and lymphoma cells [32, 33]. In esophageal cancer cells, when β-catenin signaling was inhibited, FZD1 expression was upregulated, which reversed EMT [34]. The effects of FZD1 on the extracellular matrix (ECM) play a role in EMT. WNT/β-catenin signaling promotes transcription of matrix metallopeptidase (MMP) proteins known for breaking down the ECM and decreasing cell adhesion. In breast cancer, nestin, an ECM intermediate filament protein, inhibits FZD1 expression and β-catenin signaling. This inhibition halts proliferation and invasion by decreasing the expression of MMP proteins [35]. Furthermore, a novel WNT signal sequestering mechanism by overexpression of FZD1 on the surface of glioblastoma cells activates cJun N-termianl kinase (JNK), which in turn promotes the transcription of MMP proteins [36]. FZD1 is methylated early in prostate cancer initiation, leading to loss of expression, suggesting a potential context-specific tumor suppressive role [37]. Fzd1 presents both oncogenic and tumor suppressive properties and preclinical results suggest that drugs targeting Fzd1 could be effective chemoprevention agents in the right tissue type. (Frizzled receptor roles in cancer EMT are summarized in Table 1).

### Frizzled 2

FZD2, located on chromosome 17q21.1, is a seven-transmembrane protein with a C-terminal Ser/Thr-Xxx-Val motif, and most widely known for its role in cell differentiation. Under normal physiologic conditions, FZD2 can be found in the fetal lung, brain and kidney, as well as the adult heart [26, 38]. Recently, FZD2 upregulation of tumorigenic processes has been associated with non-canonical pathways via signal transducer and activator of transcription 3 (STAT3) following WNT5a, WNT7a, or WNT3 binding [39, 40]. Through non-canonical signaling, FZD2 induces EMT, promotes migration, increases cell motility, and stimulates tumorigenesis in various cancers, highlighting its potential to be a target for chemoprevention [41]. FZD2 contributes to EMT via non-canonical signaling in hepatocellular carcinoma in vitro. Not only does FZD2 overexpression increase mesenchymal markers such as n-cadherin, vimentin, and fibronectin, but inhibition of FZD2 also decreased STAT3 activity, leading to decreased expression of STAT3’s downstream targets such as IL2RG, STAM, PLAU, SERPINE1, and MMP-3 [42]. FZD2 expression also correlates positively with mesenchymal markers snail, slug, and vimentin, as well as a inversely with epithelial marker e-cadherin in human oral squamous cell carcinoma (OSCC) [38]. This study also found that FZD2 increases cancer cell motility, migration, and invasion by increasing mesenchymal proteins and MMP-2, MMP-9, and MMP-13 [38]. FZD2 and its ligand, WNT5a, upregulate the transcription of hundreds of EMT genes necessary for transforming growth factor β receptor 1 (TGF-β), hedgehog, and notch signaling [41]. A comparison between FZD2 knockdown and overexpression found that 889 genes varied in expression, many of which were identified to induce EMT, extracellular-matrix organization, cell adhesion, angiogenesis, and morphogenesis [41]. FZD2 is highly expressed in premalignant lesions preceding esophageal adenocarcinoma but is decreased in colorectal adenomas compared to normal tissue, suggesting FZD2 could be targeted differently to intercept early lesions depending on the tissue type [43]. The minimal expression of FZD2 in normal adult tissues, along with substantial evidence of EMT promotion, highlights the chemopreventive potential of targeting FZD2.

### Frizzled 3

FZD3 is mapped on chromosome 8p21 and is expressed in various tissues such as skeletal, muscle, kidney, pancreas, cerebellum and cerebral cortex [44]. The neural crest like cell population exhibits a robust amount of FZD3 [45] and mice with FZD3 knockout die within 30 min after birth as a result of neural defects [46]. FZD3 is associated with many non-cancerous diseases like schizophrenia, Hirschsprung disease, polycystic ovarian syndrome (PCOS), and polycystic kidney disease [46–49]. In mesenchymal stem cells, which are responsible for tissue regeneration and repair, FZD3 is upregulated and causes cells to differentiate depending on which WNT ligand binds it. For example, WNT4 binding enhances osteogenic differentiation, while WNT3 and WNT5 binding promote neurogenesis and improved neurocognitive function [50, 51]. In breast cancer, the binding of WNT5 to overexpressed FZD3 inhibited the formation of filopodia, migratory organelles related to actin dynamics and EMT [52]. In both melanoma and non-melanoma cancer cell lines, FZD3 is also overexpressed, and binding of WNT5 causes the cell to project and migrate [53, 54]. Normally, secreted frizzled related protein 1 (SFRP1) interferes with WNT binding to FZD3 receptors. In a myelodysplastic syndrome (MDS) cell line, SFRP1 is transcriptionally inactivated due to epigenetic modification, allowing increased WNT ligand binding to FZD3. This hyperactivates downstream EMT genes in MDS, leading progression to acute myeloid leukemia (AML) [55]. In adenomas from early colorectal tumorigenesis, FZD3 is overexpressed, indicating a potential role for chemopreventive interventions [56],. Overexpression or activation of FZD3 in several cancer types contributes to EMT and could be a target for chemoprevention strategies.

### Frizzled 4

FZD4 is located at chromosomal region 11q14.2 and is the only frizzled protein that interacts with the angiogenic protein, norrin. Norrin-FZD4 interaction is primarily observed in retinal angiogenesis, linking FZD4 mutations to familial exudative vitreoretinopathy (FEVR) [57]. However, FZD4 also plays a role in tumor progression, migration, and invasion [58]. The role of FZD4 in EMT was first identified in patients suffering from penetrating Crohn’s disease, a common risk factor for CRC [59]. Penetrating Crohn’s disease patients have elevated levels of WNT2b and FZD4, as well as n-cadherin and vimentin, suggesting FZD4 contributes to EMT in the colon [60]. In glioblastoma stem cells (GSC), FZD4 increased nuclear β-catenin, nestin, snail, and vimentin while decreasing e-cadherin and CD133 levels [61]. In this system, FZD4-induced WNT signaling is primarily at the invasive, exterior portion at the tumor suggesting FZD4 plays a role in tumor initiation and metastasis [61]. In prostate cells, downregulation of FZD4 expression by miR-377 increased epithelial markers e-cadherin and zonula occludens–1, while decreasing levels of mesenchymal marker ets proto-oncogene-1 [62]. The ability of FZD4 to induce EMT via the TCF/LEF/WNT/β-catenin pathway in prostate cells is further supported by FZD4 knockdown causing an increase in β-integrin and e-cadherin expression, reversing EMT, and inducing cell adhesion [63]. FZD4 also contributes to EMT in non-small cell lung cancer (NSCLC) both in vitro and in vivo by activating the TCF/LEF/WNT/β-catenin pathway [58]. Although studies in premalignant lesions are minimal, FZD4 is known to play a role in mesothelial hyperplasia and Crohn’s disease, a risk factor for colorectal carcinoma [59, 64]. The presence of FZD4 in premalignant tissues along with the ability of FZD4 to induce cancer cell EMT highlights FZD4’s potential in future chemopreventive studies.

**Table 1:** Status of FZDs associated with EMT in cancer

	Cancer	Frizzled Status
FZD1	Neuroblastoma [32]	Downregulated
	Glioblastoma [36]	Receptor accumulation
	Lymphomas [33]	Downregulated
	Colorectal cancer [65]	Increased copy number
	Esophageal cancer [34]	Upregulated
	Breast cancer [35]	Downregulated
FZD2	Human oral squamous cell carcinoma [38]	Upregulated
	Hepatocellular carcinoma [39]	Upregulated
	Esophageal adenocarcinoma [66]	Upregulated
	Colorectal adenomas [66]	Downregulated
FZD3	Myelodysplastic syndrome [55]	Upregulated
	Melanoma [54]	Upregulated
	Breast cancer [52]	Upregulated
	Colorectal cancer [56]	Upregulated
	Non-melanoma [53]	Upregulated
FZD4	Prostate [62]	Upregulated
	Colorectal Cancer [60]	Upregulated
	Non-small cell lung cancer [58]	Upregulated
FZD5	Endometrial adenocarcinoma [67]	Downregulated
	Gastric cancer [68]	Downregulated
	High grade serous ovarian cancer [69]	Downregulated
	Prostate [70]	Upregulated
	Hepatocellular carcinoma [71]	Upregulated
	Acute Myeloid Leukemia [72]	Downregulated
	Ovarian cancer [73]	Upregulated
FZD6	Colorectal cancer [74]	Upregulated
	Gastric cancer [75]	Upregulated
	Breast cancer [76]	Upregulated
	Chronic lymphocytic leukemia [77]	Upregulated
	Prostate cancer [78]	Downregulated
	Glioblastoma [79]	Upregulated
FZD7	Esophageal cancer [80]	Upregulated
	Hepatocellular carcinoma [81]	Upregulated
	Gastric cancer [82]	Upregulated
	Breast cancer [83, 84]	Upregulated
	Ovarian cancer [85]	Upregulated
	Cervical cancer [86]	Upregulated
	Melanoma [87]	Upregulated
	Osteosarcoma	Upregulated
	Glioblastoma [88]	Upregulated
FZD8	Non-small cell lung cancer [89]	Upregulated
	Head and neck squamous sarcinoma [90]	Upregulated
	Gastric cancer [91]	Upregulated
	Breast cancer [92]	Upregulated
	Renal cell carcinoma [93]	Upregulated
	Colorectal cancer [94]	Upregulated
	Prostate cancer [95]	Upregulated
FZD9	Non-small cell lung cancer [24, 96]	Downregulated
	Astrocytoma [97]	Upregulated
	Acute Myeloid Leukemia [98]	Downregulated
	Hepatocellular carcinoma [99]	Upregulated
	Osteosarcoma [100]	Upregulated
FZD10	Colorectal cancer [101, 102]	Upregulated
	Renal cell carcinoma [103]	Upregulated
	Synovial sarcomas [104]	Upregulated
	Breast cancer [105]	Upregulated
	Gastric cancer [101, 106]	Upregulated
	Lung cancer [107]	Downregulated
	Esophageal squamous cell carcinoma [108]	Upregulated

### Frizzled 5

FZD5 is mapped on chromosome 2q33.3-q34 and highly expressed in fetal liver and adult pancreas while also moderately expressed in fetal kidney and adult liver [109]. FZD5 interacts with WNT2-7a,b, WNT9b, WNT-10b and WNT-11 and exerts a range of influence on cancer progression [110]. Fzd5 acts in a tumorigenic nature in ovarian cancer, as increased FZD5 expression is associated with increased expression of ECM components related to EMT processes, such as fibronectin and vitronectin, while the loss of FZD5 presented a decrease in the ECM components [73]. In hepatocellular carcinoma (HCC), YTH domain containing family protein-1 (YTHDF1) facilitates EMT in part by increasing translation of FZD5 and activating canonical β-catenin signaling [111]. In contrast, in endometrial adenocarcinoma FZD5 is downregulated compared to atrophic endometrium, which may suggest a tumor suppressive role in some cancers [67]. This is also supported in gastric cancer where the epithelial factor ELF3 cooperates with FZD5 to block EMT signaling [68]. In high grade serous ovarian cancer (HGSOC), loss of ELF3, LRG4, and FZD5 disrupts the epithelial phenotype [69]. In prostate cancer, FDZ5 is required for the anti-proliferative effect of WNT5a in vitro [70]. FZD5 is methylated early in the development of AML and FZD5 antibodies have been used to inhibit receptor activity in pancreatic and CRC cell lines, suggesting the potential for targeting FZD5 in AML and other cancers for prevention [72, 112]. The variation of Fzd5 activity between different cancers by either promoting or suppressing EMT and tumorigenesis presents an opportunity as a potential, specific drug target for both premalignant and malignant tissues.

### Frizzled 6

FZD6 is located on chromosome 8q22.3-q23.1 and does not contain a second C-terminal PDZ domain-binding motif present in other FZD receptors [113]. In many cancers FZD6 receptor is overexpressed, however multiple studies have observed a downregulation of FZD6 receptor as a suppressor of cancer [11, 77, 114–116]. In breast cancer cells, FZD6 expression induces EMT, while knockout of FZD6 inhibits motility and invasiveness [76]. In mice, FZD6 was upregulated in preleukemic cells and is required for malignant transformation to chronic lymphocytic leukemia (CLL) [77]. In mesenchymal glioblastoma, FZD6 is overexpressed, inducing a mesenchymal phenotype through the CaMKII-TAK1-NLK pathway [79]. NPTX2, an extracellular ligand that binds to NPTX receptor, was overexpressed in patients with metastatic colorectal cancer and interacted with FZD6 to promote invasion [74]. FZD6 has increased expression in colorectal adenomas compared to normal tissue [117]. In contrast, in gastric cancer cells where *H. pylori* increases β-catenin activity, the breast cancer chemoprevention agent tamoxifen combined with 17β-Estradiol increased FZD6 expression and repressed gastric cancer tumorigenesis [9]. In prostate cancer, FZD6 is down regulated, leading to an increase in the stem phenotype of prostate cancer cells. When treated with luteolin, a dietary flavonoid with anti-cancer activity, FZD6 expression increased, and the stem phenotype decreased [78]. FZD6 may present a unique scenario among FZD receptors, where it contributes to both promotion and suppression of EMT depending on the cancer type and could be a target for highly personalized delivery of chemoprevention.

### Frizzled 7

FZD7 is located at chromosome 2q33 and shares 97% homology with FZD1 and FZD2 [30]. During development FZD7 is known to have role in neural crest induction, gastrulation, and intestinal homeostasis [30]. Overexpression of FZD7 is associated with EMT in many types of cancer. In melanoma, glioblastoma, and osteosarcoma, FZD7 expression is increased and inhibition of FZD7 suppresses EMT [88, 118, 119]. In cervical cancer, inhibiting FZD7 reverses EMT through decreased MMP2 and MMP9 activity [86]. In esophageal squamous cell carcinoma, overexpression of FZD7 in the presence of WNT3a leads to an increase in downstream EMT target MMP7; the depletion of FZD7 inhibited EMT [80]. In ovarian cancer, over expression of FZD7 along with twist1 promotes EMT [85]. In gastric cancer, FZD7 promotes EMT through canonical WNT signaling and silencing of FZD7 inhibits EMT [82]. In hepatocellular carcinoma cells, FZD7 binds with Cripto1, LRP6, and DVL3 to promote EMT and stemness [81]. In breast cancer, γ-tocotrienol, a vitamin E compound, acts as a chemopreventive agent by reducing the expression of FZD7, increasing expression of canonical WNT signaling inhibitors, and reversing EMT [83]. FZD7 is overexpressed in triple negative breast cancer and interference with FZD7 signaling in this context reduces the EMT induced by WNT/β-catenin signaling [84]. When FZD7 is inhibited by an antibody coated nanoshell in breast cancer cells, the EMT phenotype is reduced, identifying FZD7 as targetable in cancer cells and offering a potential delivery approach for FZD7 chemoprevention [120].

### Frizzled 8

FZD8 is located on the chromosomal region 10p11.2 and is primarily found in fetal kidney and brain tissue, but also in adult kidney, pancreas, heart, and skeletal muscle tissues [121]. FZD8 plays a role in tumor initiation, invasion, and metastasis in various cancers, including head and neck squamous carcinoma (HNSCC), gastric, breast, NSCLC, thyroid, cervical, renal cell carcinoma (RCC), and prostate [89–91]. The most common WNT ligands that interact with the FZD8 receptor are WNT11 and WNT5b [122, 123]. FZD8 is a downstream target of multiple oncogenes including ETS-related gene (ERG) in prostate cancer, SRC in lung adenocarcinoma, and Mesenchymal–Epithelial Transition (MET) in HNSCC [90, 95, 124]. The role of FZD8 in EMT was initially described in RCC. Normally overexpressed in RCC, FZD8 upregulation increases mesenchymal markers snail and vimentin, while FZD8 knockdown reduced mesenchymal markers and increased expression of the epithelial marker e-cadherin [93]. In prostate cancer, FZD8 drives EMT through crosstalk between WNT11 and TGF-β Receptor 1 [122]. In this study, FZD8 silencing decreased migration, invasion, and cell number; mesenchymal gene expression was also reduced, including vimentin, snail, twist1, zeb1, and e-cadherin. In lung cancer, inhibition of FZD8 by a monoclonal antibody blocks e-cadherin degradation, thus preventing a transformed phenotype [124]. Silencing FZD8 with miRNAs to inhibit FZD8 activity decreased EMT associated proteins TCF4, MMP7, and nuclear β-catenin in both breast cancer and colorectal cancer [92, 125].While FZD8 has primarily been investigated in aggressive and metastatic tumors, several studies point to a role for FZD8 in premalignant lesions. FZD8 was investigated as a potential drug target to inhibit HNSCC stem-like cells in order to block tumor formation [90]. The tyrosine protein-kinase c-Met upregulated FZD8 through the Erk/c-Fos cascade to initiate interaction of c-met with the WNT/β-catenin pathway. Pharmacologic inhibition of the c-Met/FZD8 axis decreased the HNSCC stem cell population and inhibited initiation of head and neck squamous carcinomas [90]. FZD8 in overexpressed in both intestinal metaplasia and gastric cancer tissues, suggesting alterations in FZD8 may occur early in progression and making it a potential target for chemoprevention in premalignant tissues [91]. FZD8 is methylated early in the development of AML, suggesting there may be specific contexts where FZD8 loss supports cancer development and that targeted re-expression of FZD8 could be a chemoprevention strategy [72]. The prominent role of FZD8 in EMT and its potential involvement in premalignant lesion development present a favorable drug target for cancer chemoprevention.

### Frizzled 9

FZD9 is commonly expressed in brain, testis, skeletal muscle and renal tissue [99]. FZD9 is located in the chromosomal region 7q11.23, and deletions of this chromosomal region contribute to the neurological disorders of Williams-Beuren syndrome [126]. Additionally, FZD9 has an essential role in osteoblast differentiation for bone formation and pre-B cell development [127]. FZD9 has been studied in multiple cancers including NSCLC, astrocytoma, osteosarcoma, AML, and hepatocellular carcinoma (HCC) [96–99, 127]. FZD9 interacts with several WNT ligands including WNT2, WNT5a, and WNT3a depending on the tumor; these interactions promote EMT and invasiveness. In aggressive osteosarcoma, FZD9 and WNT2 expression are induced by proto-oncogene c-Fos [100]. Downregulation of c-Fos decreased WNT2 and FZD9 expression and inhibited EMT. Increased levels of FZD9 expression promotes EMT in HCC cells [99]. FZD9 is also upregulated in astrocytomas and induces EMT through the canonical WNT/β-catenin/TCF pathway by binding WNT5a [97, 128, 129].

In contrast, AML cells exhibit decreased FZD9 expression due to hypermethylation of the promoter region or genes regulating the FZD9 receptor, and treatment with demethylating agents restores FZD9 expression [98]. In NSCLC, FZD9 expression is downregulated in pre-malignant and tumor tissues and induced expression of FZD9 expression inhibits *in vitr*o lung cancer cell colony formation [8, 24]. In lung, binding of WNT7a to Fzd9 activates peroxisome proliferator activated receptor γ (PPARγ) and anti-EMT signaling, helping to maintain a normal lung epithelium [96, 130]. Iloprost is a prostacyclin analogue that requires FZD9 to activate anti-EMT signaling through PPARγ in vitro [8, 131]. Prostacyclin reduces tumor burden in vivo without the presence of its traditional receptor, suggesting that Fzd9 may be an alternative receptor for prostacyclin in the lung epithelium [131]. In a phase II clinical trial, oral Iloprost reduced endobronchial dysplasia, a precursor to squamous cell carcinoma, and is currently being studied with inhaled [132]. Cigarette smoke exposure downregulates FZD9 in cell and mouse models, and this is reversed in response to iloprost in vitro [8, 24]. WNT7a/FZD9 signaling inhibits EMT in NSCLC cell lines, suggesting that reactivation of FZD9 signaling could slow lung lesion progression [96]. Changes in the FZD9 receptor in cancer highlight it as a potential target for precision prevention of tumor development, depending on the tissue targeted and a specific drug’s mechanism of action.

### Frizzled 10

FZD10, located in the chromosomal region 12q24.33, plays a role in cell migration, cell polarity, cell adhesion, neural patterning, and embryogenesis. FZD10 is highly expressed throughout embryonic development, however there is minimal to no expression in developed organs [101]. FZD10 expression has been studied in multiple cancers due to activation of both canonical and non-canonical WNT signaling and EMT. FZD10 upregulation was first identified in synovial sarcomas, where it enhances phosphorylation of disheveled proteins 2/3 (DVL2/3), upregulating downstream targets Rac1 and JNK [104]. The upregulation of JNK and Rac1 increased actin cytoskeleton reconstruction and anchorage-independent growth, both characteristics of EMT [133]. In renal cell carcinoma, binding of hypoxia-inducible protein 2 to FZD10 activates β-catenin EMT signaling, and this occurs in early-stage patient samples [103]. In breast cancer, epigenetic silencing of Fzd10 by BRMS1L inhibits tumor progression and metastasis. BRMS1L knockdown increased levels of expression of FZD10 and induced EMT [105]. This mechanism also functions in esophageal squamous cell carcinoma [108]. In colorectal cancer (CRC), cells with high FZD10 expression were more efficient than cells with low FZD10 at stimulating EMT [134]. In progression of CRC, FZD10 expression increases from non-dysplastic to dysplastic tissue, suggesting it is activated early in CRC development [102]. In melanoma tissues, FZD10 expression is elevated in dysplasia but decreases with advanced stage of tumors, indicating that its key effects may be to initiate and support progression of premalignant lesions [102]. In gastric cancer, FZD10 expression is elevated compared to normal tissue and also declines slightly as tumors advance [102, 106]. In esophageal stem cells, FZD10 expression is high, suggesting FZD10 may play a role in esophageal tumor initiation [135]. In contrast to most other studies, one study of genome-wide methylation in lung cancer found that FZD10 is hypermethylated and downregulated in tumors compared to normal tissue and FZD10 has low expression in medulloblastomas compared to normal cerebellum [107, 136].The presence of FZD10 expression in early lesions of multiple cancers but lack of expression in normal adult tissues highlight a key characteristic of chemoprevention targets. Combined with its likely role in EMT process, this makes FZD10 a favorable drug target for prevention.

**Table 2:**
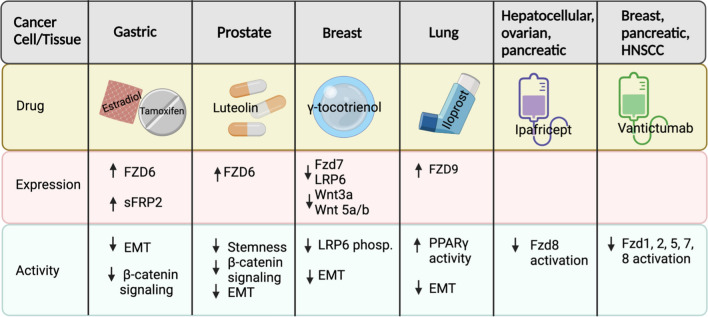
Drugs with effects on Frizzleds in cancer cell lines or tissues

### Frizzleds in cancer chemoprevention

Cancer chemoprevention is a developing field with the potential to have significant health impacts for populations at high risk of cancer [137]. The characterization of potential targets for prevention agents is a critical area of research. FZDs represent a family of receptors with varying roles across cancer types that could be new targets for cancer chemoprevention. While other G-protein coupled receptors are targeted by drugs in many clinical trials, FZD receptors have not historically been considered appealing as direct drug targets [138]. Recent evidence demonstrates, however, that small molecule ligands can actually target the transmembrane core of FZD receptors [17]. This data supports continued work on studies such as those investigating Fzd9 as a direct agonistic target of iloprost, a lung cancer chemoprevention drug with EMT targets that has had success in a phase II clinical trial [8, 24, 25, 132]. Other approaches to FZD targeting may also be developed into prevention strategies (Table 2). Antibodies have been used to inhibit both individual or multiple FZD receptors at conserved domains [120, 139]. The prevalence of antibody approaches in cancer treatment could benefit the development of prevention agents by establishing toxicity and delivery methods that would inform prevention studies. RNAi approaches for inhibiting FZD receptors are plentiful and the rapidly improving application of these therapies may also prove valuable for prevention [140]. The use of agents that target FZDs will need to account for potential compensatory activity of FZD receptors or effects on multiple FZDs due to highly conserved receptor sequences. Identification of drug mechanisms and markers is a persistent challenge of chemoprevention agent development and these will also be factors for any agents targeting FZDs.

A role for Frizzled receptors in early tumor progression has been identified in multiple studies. A Fzd2 antibody inhibited tumor growth in a dose dependent manner in HCC xenograft models [42]. FZD3 is tumorigenic in colorectal cancer development, as 75% of colorectal polyps, 89% of colorectal adenomas, and 100% of colorectal cancer specimens expressed FZD3 [56]. FZD10 expression is positively correlated with colon cancer progression, from hyperplasia to metastatic tissues [102]. Similarly, upregulation of Fzd4 has been identified not only in malignant pleural mesothelioma, but also mesothelioma hyperplasia [64]. FZD5 and FZD8 have roles in early development in both pancreatic cancer and acute myeloid leukemia [72, 112]. In contrast, Fzd9 has anti-tumor function through activation of the PPARγ pathway in the lung, which prevents development of early lesions, and is required for activity of the chemoprevention drug iloprost [8, 24, 131]. A phase II clinical trial found that Iloprost, a prostacyclin analog, improved early lung lesions in former smokers without significant adverse effects [132]. While the role of FZD receptors in tumor progression has been predominantly studied in advanced tumors, there is clear potential for targeting Frizzled proteins in premalignant tissues as a chemoprevention strategy.

Chemoprevention studies have frequently targeted EMT programming to reverse progression of premalignant lesions. Metformin has been explored as a preventive agent for many cancers, including colorectal, lung, and breast, and has effects on EMT, in part through a reduction in WNT/β-catenin signaling [20]. Chemoprevention with silibinin has been used in colorectal and prostate models, where it attenuates EMT through changes in gene expression and effects on components of the tumor microenvironment [21, 141]. In gastric cancer, β-carotene reduced EMT caused by tobacco exposure in mice [23]. The anti-inflammatory drug celecoxib has cancer preventive activity by reducing EMT in colon, bladder, and breast cancer cells through its effects on WNT signaling and the microenvironment [22, 142, 143]. Chemoprevention targets premalignant lesions, and data on the role of EMT in the progression of premalignant lesions to cancer is steadily increasing. In dysplastic leukoplakia, a precursor to oral squamous cell carcinoma, EMT markers are elevated compared to non-dysplastic leukoplakia or normal tissue [144]. Infection of mice with *H.pylori* leads to premalignant lesions in the gastric mucosa that have EMT phenotypes [145]. EMT gene expression is elevated in blood samples from tobacco exposed patients with premalignant head and neck squamous cell carcinoma lesions [146]. In premalignant cervical lesions, EMT facilitates progression from low to high grade lesions [147]. In lung cancer, EMT gene expression changes with exposure to cigarette carcinogens and correlates with the development of early adenomas in mice [25]. FZDs have a strong connection to EMT through activation of WNT/β-catenin signaling, which generally increases progression of premalignant lesions to cancers. Some context specific FZD activity does the opposite, leading to the reversal of EMT through inhibition of canonical WNT/β-catenin signaling or activation of non-canonical pathways.

While there has not yet been a clinical study that targets the Wnt/β-catenin pathway in premalignant tissues, the pivotal role Wnt/β-catenin signaling plays in tumorigenesis and cancer progression has led to more than twenty clinical trials with drugs that manipulate Wnt/β-catenin signaling [148]. In order to identify the most efficient way to abrogate oncogenic Wnt/β-catenin signaling, there are phase I clinical trials investigating drugs that target pathway components, including Dickkopf-1, Porcupine Acetyltransferase (PORCN), CBP/β-catenin complex, B-catenin gene expression, or FZD receptors [148]. Ipafricept (OMP-54F28) is a FZD8 decoy receptor that binds WNTs and has been tested as both a monotherapy in solid tumors and dual therapy for HCC, ovarian cancer, and pancreatic cancer [148, 149]. Vantictumab (OMP-18R5), a monoclonal antibody against FZD receptors, has been studied as a monotherapy and dual therapy in clinical studies for metastatic breast cancer, pancreatic cancer, and HNSCC [150–153]. While these drugs maintain disease stability or reverse tumor progression, fragility fractures were a common side effect. Compensating for bone loss helps with toxicity in therapeutic trials, but inhibiting specific FZDs with a minimal role in bone maintenance would be a more tolerable option for chemoprevention. Cancer treatment drugs are often not ideal chemoprevention drugs due to off-target effects and cost, however, demonstrated ability to manipulate FZD pathways is promising for future investigation of chemoprevention approaches.

## Conclusion

The development of cancer chemoprevention agents lags far behind the development of cancer chemotherapies, but based on identification of a role in the early progression of several types of cancer, FZD receptors could be candidates for chemoprevention approaches. The frequent association of FZD receptors with EMT, which contributes to cancer development at all stages, also suggests FZDs could be targets for preventing early lesion progression, metastasis, or recurrence. The current use of FZD targeting drugs to stabilize or regress tumors suggests feasibility of also targeting FZD receptors to intercept premalignant and early lesions. Chemoprevention approaches using FZD agonists or antagonists will require context specific application, along with precision medicine approaches relying on prediction and response markers. Despite the research challenges, FZD receptors present an important opportunity for new drug development that could advance the application of much needed cancer chemoprevention strategies in high risk populations.

## Data Availability

Not applicable.

## Abbreviations

AML: Acute myeloid leukemia
BRMS1L: Breast Cancer Metastasis Suppressor 1 like
CLL: Chronic lymphocytic leukemia
CRC: Colorectal cancer
DAG/IP3: Diacylglycerol/Inositol Trisphosphate
DVL: Disheveled
E2: 17β-Estradiol
ECM: Extracellular matrix
EMT: Epithelial to mesenchymal transition
ERG: ETS-related gene
ESCC: Esophageal squamous cell carcinoma
FEVR: Familial exudative vitreoretinopathy
FZD: Frizzled
GPCR: G-protein coupled receptors
GRHL1: Grainyhead like transcription factor 1
GSC: Glioblastoma stem cells
HCC: Hepatocellular carcinoma
HGSOC: High grade serous ovarian cancer
HNSCC: Head and Neck Squamous Sarcinoma
JNK: C-Jun N-terminal kinase
LRP: Low-density lipoprotein receptor related proteins
MDS: Myelodysplastic syndrome
MET: Mesenchymal–epithelial transition
MMP: Matrix metallopeptidase
NPTX2: Neuronal pentraxin 2
NSCLC: Non-small cell lung cancer
OSCC: Oral Squamous cell carcinoma
PCOS: Polycystic ovarian syndrome
PCP: Planar cell polarity
PPARγ: Peroxisome proliferator activated receptor γ
RCC: Renal cell carcinoma
ROR2: Receptor tyrosine kkinase like orphan receptor 2
RYK: Receptor-like tyrosine kinase
SFRP1: Secreted frizzled related protein 1
STAT3: Signal transducer and activator of transcription 3
TCF/LEF: T-cell Factor/Lymphoid Enhancer Factor
TGF-β: Transforming growth factor β
VANGL: Vanglike-1
WNT: Wingless/Int
YTHDF1: YTH domain containing family protein-1
